# A Rare Case of Metastatic Adenocarcinoma Masquerading as Disseminated Tuberculosis

**DOI:** 10.7759/cureus.71263

**Published:** 2024-10-11

**Authors:** Swapnil N Deshmukh, Vasu Agarwal, Sidhaant Nangia

**Affiliations:** 1 Respiratory Medicine, Dr. D Y Patil Medical College, Hospital and Research Centre, Dr. D Y Patil Vidyapeeth (Deemed to be University), Pune, IND

**Keywords:** adenocarcinoma, disseminated, intestine, lung, obstruction, tuberculosis

## Abstract

A 68-year-old female with no known comorbidities presented with a three-month history of abdominal pain, nausea, vomiting aggravated by food intake, dry cough, gastro-oesophageal reflux disease, breathlessness, low-grade fever, and significant weight loss. Initial investigations including a plain radiograph of the erect abdomen and contrast-enhanced computed tomography abdomen showed irregular concentric thickening of the large bowel along with proximal dilation of small bowel loops which was suggestive of subacute intestinal obstruction secondary to abdominal tuberculosis (TB). The patient also complained of persistent dry cough for which a chest radiograph and computed tomography (CT) thorax were done which showed features suggestive of pulmonary TB. Conservative management was initiated for subacute intestinal obstruction but persistent cough led to further evaluation with bronchoscopy and transbronchial lung biopsy, revealing invasive mucin-secreting adenocarcinoma. A subsequent PET-CT scan confirmed a large mass in the ileocecal region causing obstruction, multiple iliac lymph nodes, pancreatic and skeletal deposits, and lung opacities indicative of lymphangitis carcinomatosis. Despite the recommendation for exploratory laparotomy, the patient opted for conservative management due to her age and associated risks. This case highlights the importance of clinical symptoms and signs mimicking disseminated TB. Concomitant presence of chronic diseases with overlapping symptoms can lead to diagnostic dilemmas.

## Introduction

Tuberculosis (TB) remains a significant global health issue, particularly in countries like India, which bear a substantial burden of the disease. According to the Global TB Report 2022, an estimated 10.6 million incident cases of TB were reported in 2021 [1]. Abdominal TB, while relatively rare, accounts for about 12% of extrapulmonary TB cases and is increasingly recognized in both developing and developed countries [2] and it poses diagnostic challenges due to its non-specific symptoms. Mucinous adenocarcinoma form 5% of all lung carcinomas [3]. They are mucin-producing tumors, most commonly presenting in smokers in the 55-65-year age group [4]. This case report presents a unique instance where metastatic adenocarcinoma mimicked disseminated TB, complicating the diagnostic process. A 68-year-old female, initially treated for presumed abdominal and pulmonary TB based on clinical and radiological findings, was later diagnosed with invasive mucin-secreting adenocarcinoma through further evaluation. This case underscores the necessity for a high index of suspicion for malignancies in patients presenting with TB-like symptoms, especially in TB-endemic regions.

## Case presentation

**Figure 1:**
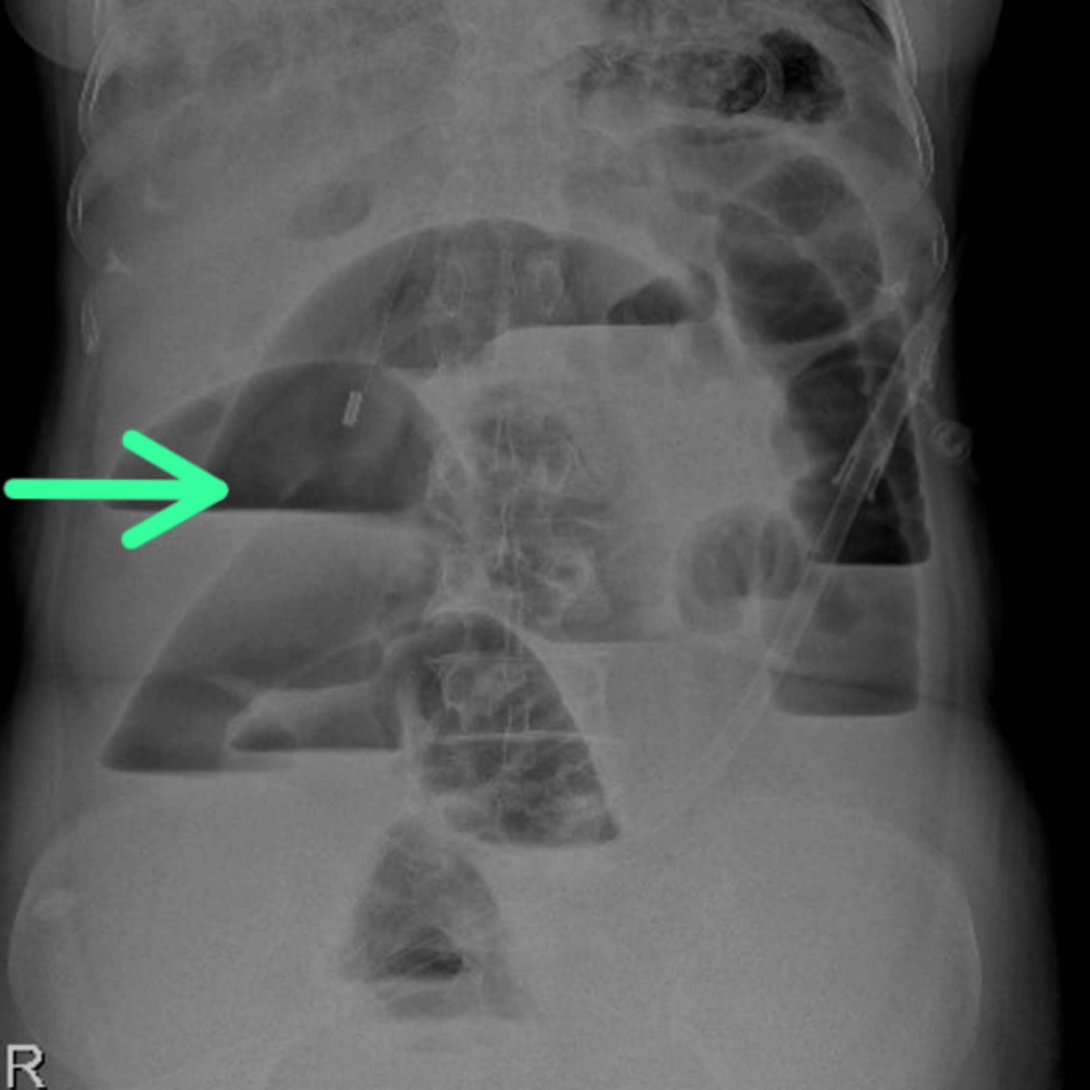
Radiograph of the Erect Abdomen Showing Multiple Air Fluid Levels

A 68-year-old female patient presented to the surgical department with a three-month history of abdominal pain, nausea, vomiting, low-grade fever, significant weight loss, and reduced appetite. Nausea and vomiting were aggravated post-food consumption. Suspecting an abdominal obstruction, a plain radiograph of the erect abdomen was taken, which suggested multiple air fluid levels in the abdomen and further computed tomography (CT) of the abdomen indicated irregular concentric thickening of the large bowel along with enlarged abdominal lymph nodes (Figure 1).

**Figure 2:**
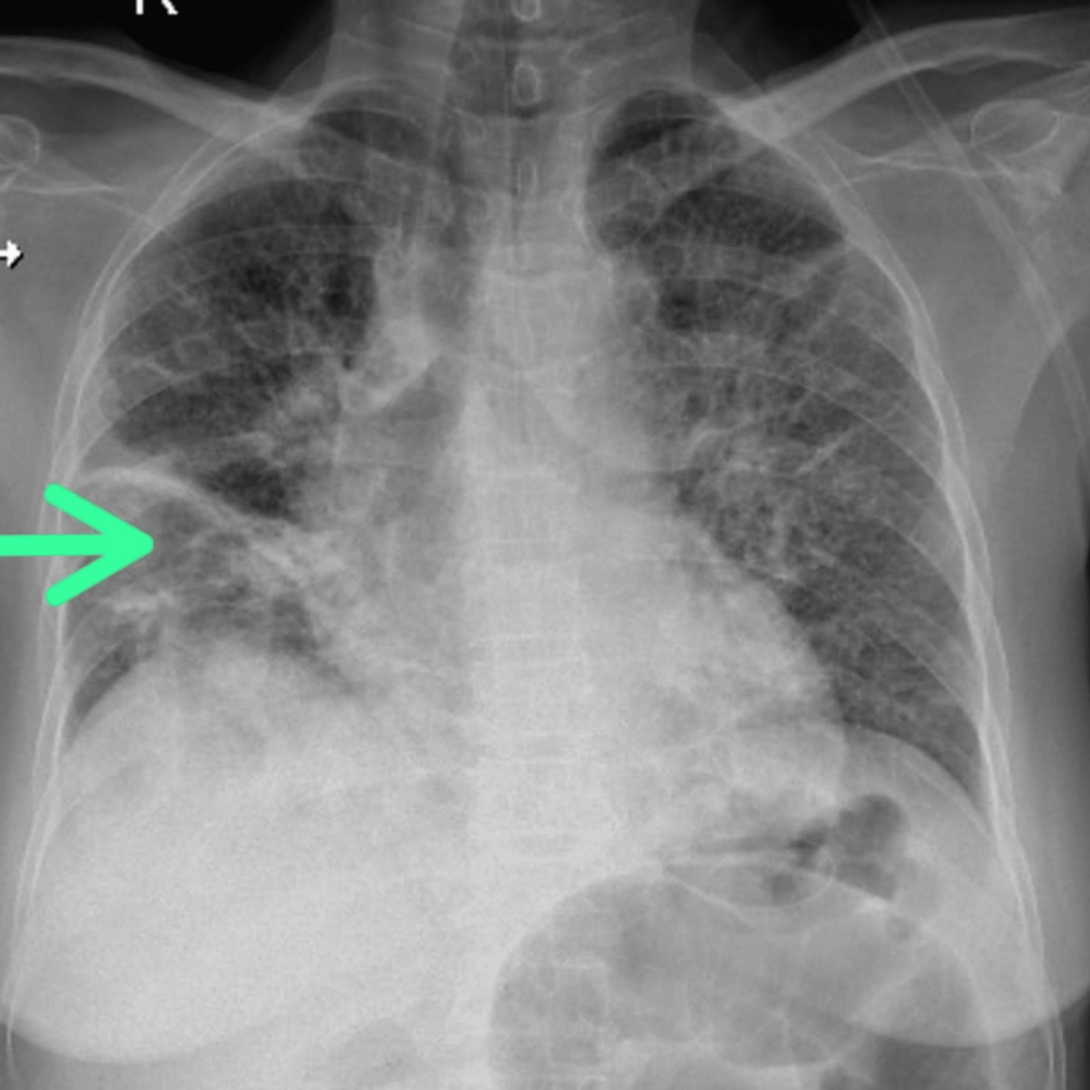
Plain Radiograph of the Chest

**Figure 3:**
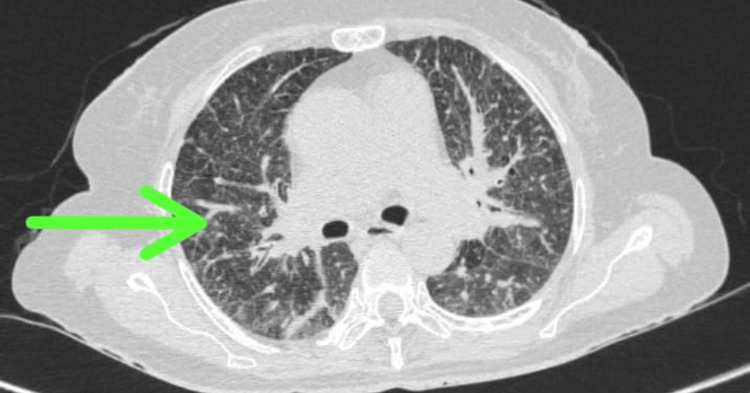
High-Resolution CT of the Chest

Symptoms, signs, and radiology were suggestive of subacute intestinal obstruction, most likely due to abdominal TB. The patient also had respiratory complaints like dry cough and breathlessness; hence to rule out disseminated TB, a chest radiograph and chest CT were done which showed areas of consolidation in bilateral lung with bilateral ground glass opacities. There were multiple tiny round smoothly marginated pulmonary nodules in the peripheral region of the antero-medial and superior segment of the left lower lobe and superior segment of left lower lobes (Figures 2, 3).

The patient received prophylactic intravenous antibiotics, and non-steroidal anti-inflammatory drugs for abdominal pain and received a liquid diet via a nasogastric tube. The patient was transferred to the respiratory ward for bronchoscopy in view of suspected pulmonary TB.

**Figure 4:**
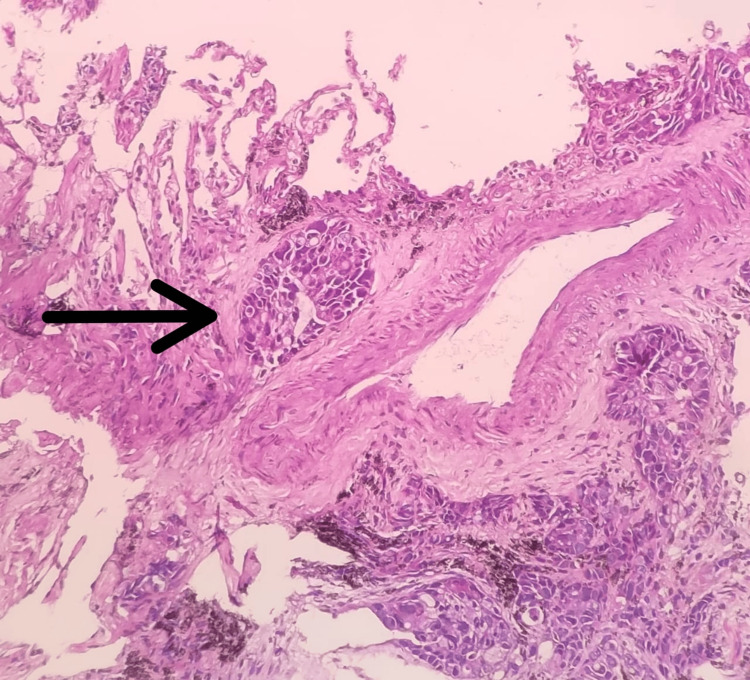
Histopathological Image Showing Nests of Malignant Cells Around the Broncho-vascular Bundle

The results of the bronchoalveolar lavage (BAL) samples were negative for any microbiological evidence of TB. Transbronchial lung biopsy was planned to reach a definitive diagnosis which revealed invasive mucin-secreting adenocarcinoma on histopathology. It showed alveolated lung parenchyma with solid nests of moderately pleomorphic, malignant epithelial cells with vacuolated cytoplasm, eccentric nuclei, and prominent nucleoli. Intracytoplasmic mucin was present (Figure 4).

A PET-CT scan was recommended by a medical oncologist and the results showed that there was a large mass lesion in the ileocecal region that was causing obstruction of the small bowel along with multiple iliac lymph nodes in the abdomen, pancreatic and skeletal deposits, and multiple ground-glass opacities in both the upper and lower lungs on both sides, along with interstitial and peri-bronchial septal thickening, which is indicative of lymphangitis carcinomatosis. A surgical opinion recommended an exploratory laparotomy to alleviate the abdominal symptoms. However, the patient decided against undergoing surgery and instead opted for conservative care. This decision was made due to the poor surgical outcome owing to the patient's age and poor prognosis of the case. The patient refused further management, including molecular studies for targeted therapy and chemotherapy, due to financial constraints and left the hospital against medical advice.

## Discussion

Mucinous adenocarcinoma, a mucus-producing lung cancer, affects 5% of lung cancer patients, mainly smokers aged 55-65 with a varied clinical presentation. Radiologically, it presents with consolidation, some ground glass opacities, and enlarged lymphadenopathy [5]. Diagnosis is mainly done by biopsy. Due to the vague clinical and radiological presentation, there is a need for lung tissue biopsy. In endemic countries such as India, the first differential diagnosis of such presentation will be TB. TB can have a variety of radiological presentations like upper lobe consolidation, miliary opacities, enlarged lymphadenopathy, pleural effusion, and bronchogenic pneumonia [6]. These presentations are also similar to adenocarcinoma. TB can affect multiple organs simultaneously. In abdominal TB, the bowel often thickens irregularly, causing abdominal pain and vomiting. Our patient's symptoms, combined with chest X-ray findings, strongly suggested widespread TB due to its high prevalence in the region. There were no risk factors like smoking or secondhand smoke to indicate adenocarcinoma at the beginning. It would have been treated as a case of disseminated TB or community-acquired pneumonia if not for bronchoscopy and biopsy. Biopsy with immunohistochemistry is highly specific and sensitive for diagnosis of adenocarcinoma [7]. It is thus with biopsy we could differentiate adenocarcinoma from pulmonary TB. The gravity of the case lies in the fact that since both the lungs were affected, the patient already was in the last stages of adenocarcinoma with a poor prognosis. Further molecular testing is required to establish a specific treatment regimen. A review article conducted over a period of three years enrolled 2908 patients who were referred to rule out neoplasms in suspected pulmonary infections. Out of these, the majority of patients 2713 (93.3%) were found to have a neoplastic process and only 37 (1.3%) had an infection [8]. A study by Khan et al. concluded that adenocarcinoma of the lung may be considered a rare but sinister differential diagnosis of miliary shadows on chest imaging [9]. Similar findings were also reported in a case in Turkey [10]. On the other hand, making adenocarcinoma one of the first differential diagnoses is difficult owing to the heavy expenditure on a battery of tests. Patients presenting with constitutional symptoms like cough, fever weight loss, and opacities on chest radiology should first undergo microbiological testing for TB, either via sputum analysis or BAL for patients unable to expectorate. Along with BAL, lung biopsy can be an effective tool to differentiate between inflammation, infection, and carcinoma and thus should be routinely performed with bronchoscopy. This can thus help in reducing the time of diagnosis and allow timely management and targeted therapy for the underlying disease.

## Conclusions

This rare case of metastatic adenocarcinoma masquerading as disseminated TB highlights the complexities and challenges of diagnosis in regions where TB is extremely prevalent. Because TB and adenocarcinoma are similar clinically and radiologically, thorough examinations involving biopsy and imaging are required to avoid misdiagnosis. When elderly patients arrive with uncommon presentations of TB, doctors should maintain a high index of suspicion for malignancies in order to ensure a clear diagnosis and appropriate therapy. This case emphasizes how important it is to employ comprehensive diagnostic methods to differentiate between infectious and malignant illnesses, which will eventually improve patient outcomes.
